# Loss of Olfactory Receptor Genes Coincides with the Acquisition of Full Trichromatic Vision in Primates

**DOI:** 10.1371/journal.pbio.0020005

**Published:** 2004-01-20

**Authors:** Yoav Gilad, Victor Wiebe, Molly Przeworski, Doron Lancet, Svante Pääbo

**Affiliations:** **1**Max Planck Institute for Evolutionary AnthropologyLeipzigGermany; **2**Department of Molecular Genetics, Weizmann Institute of ScienceRehovotIsrael

## Abstract

Olfactory receptor (OR) genes constitute the molecular basis for the sense of smell and are encoded by the largest gene family in mammalian genomes. Previous studies suggested that the proportion of pseudogenes in the OR gene family is significantly larger in humans than in other apes and significantly larger in apes than in the mouse. To investigate the process of degeneration of the olfactory repertoire in primates, we estimated the proportion of OR pseudogenes in 19 primate species by surveying randomly chosen subsets of 100 OR genes from each species. We find that apes, Old World monkeys and one New World monkey, the howler monkey, have a significantly higher proportion of OR pseudogenes than do other New World monkeys or the lemur (a prosimian). Strikingly, the howler monkey is also the only New World monkey to possess full trichromatic vision, along with Old World monkeys and apes. Our findings suggest that the deterioration of the olfactory repertoire occurred concomitant with the acquisition of full trichromatic color vision in primates.

## Abstract 2

Examination of olfactory receptor genes in 19 primate species suggests that the olfactory repertoire lost complexity as our ancestors acquired full-color vision

## Introduction

Olfactory receptor (OR) genes provide the basis for the sense of smell (Buck and Axel 1991) and, with more than 1,000 genes, comprise the largest gene superfamily in mammalian genomes (Glusman et al. 2001; Zozulya et al. 2001; Young and Trask 2002; Zhang and Firestein 2002; Olender et al. 2003). OR genes are organized in clusters (Trask et al. 1998; Young and Trask 2002) and in humans are found on every chromosome save the Y and 20 (Glusman et al. 2001; Zozulya et al. 2001). On the basis of sequence similarity, they are classified into two major classes and 17 families (Glusman et al. 2001). All OR genes have an approximately 1 kb coding region that is uninterrupted by introns (Ben-Arie et al. 1994; Gilad et al. 2000).

Interestingly, approximately 60% of human OR genes carry one or more coding region disruptions and are therefore considered pseudogenes (Rouquier et al. 1998; Glusman et al. 2001; Zozulya et al. 2001). In nonhuman apes, the fraction of OR pseudogenes is only approximately 30% (Gilad et al. 2003). However, both humans and other apes have a significantly higher fraction of OR pseudogenes than do the mouse or the dog (approximately 20%) (Young et al. 2002; Zhang and Firestein 2002; Olender et al. 2003). Thus, there has been a decrease in the size of the intact OR repertoire in apes relative to other mammals, with a further deterioration in humans (Rouquier et al. 2000; Gilad et al. 2003).

Although the causes are unclear, it seems reasonable to speculate that the high fraction of OR pseudogenes in apes reflects a decreased reliance on the sense of smell in species for whom auditory and visual cues may be more important (e.g., Dominy and Lucas 2001). We were therefore interested in investigating whether the high fraction of OR pseudogenes is characteristic of primates as a whole and, if not, to pinpoint when the proportion of OR pseudogenes increased. To this end, we randomly selected subsets of 100 OR genes in 19 primate species, including a human, four apes, six Old World monkeys (OWMs), seven New World monkeys (NWMs) and one prosimian. We find that a decrease in the size of the intact olfactory repertoire occurred independently in two evolutionary lineages: in the ancestor of OWMs and apes, and in the New World howler monkey.

## Results and Discussion

**Figure 1:**
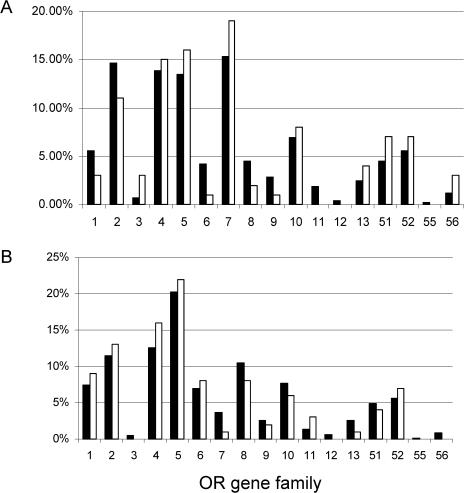
Results of the Pilot Study in Human and Mouse The percentage of OR genes from each family is given for the entire repertoire (filled bars) and a sample of 100 genes amplified using PC1 and PC2 degenerate primers (open bars). (A) OR genes in human. (B) OR genes in mouse. None of the differences between the full repertoires and the samples are significant at the 5% level. Only full-length OR genes (larger than 850 bp) were considered.

**Figure 2:**
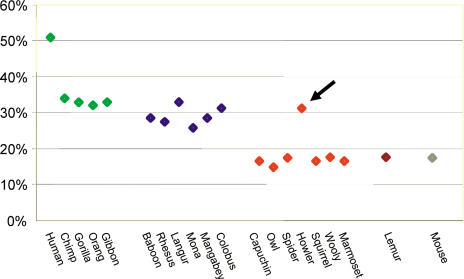
The Proportion of OR Pseudogenes in 20 Species Primate species are color-coded according to family. The arrow points to the howler monkey. Datapoints (from left to right) are for apes (green): human (Homo sapiens), chimpanzee (Pan troglodytes), gorilla (Gorilla gorilla), orangutan (Pongo pygmaeus), gibbon (Hylobates syndactylus); for OWMs (blue): Guinea baboon (Papio papio), rhesus macaque (Macaca mulatta), silver langur (Trachypithecus auratus), mona (Cercopithecus mona), agile mangabey (Cercocebus agilis), black-and-white colobus (Colobus guereza); for NWMs (red): brown capuchin monkey (Cebus apella), southern owl monkey (Aotus azarai), spider monkey (Ateles fusciceps), black howler monkey (Alouatta caraya), squirrel monkey (Saimiri sciureus), wooly monkey (Lagothrix lagotricha), common marmoset (Callithrix jacchus); for one prosimian primate (brown): crowed lemur (Eulemur mongoz); and for the mouse (Mus musculus) (grey).

Owing to the high levels of DNA sequence divergence among the primate species in our sample, orthologous OR genes could not be amplified by primers designed based on human sequences (Gilad et al. 2003). Instead, we used two sets of degenerate primer pairs, constructed to amplify OR genes from all of the species studied (see Materials and Methods). We then cloned the PCR products and determined the sequences of clones until we had identified 100 distinct OR genes from each species. A danger of this approach is that degenerate primers may bind preferentially to certain OR genes, thereby resulting in a biased representation of the OR repertoire. To safeguard against this, we tested the degenerate primers on human and mouse, for which the entire OR gene repertoire is known, by using them to amplify 100 OR genes from the two species. The sample thus obtained faithfully represented the composition of the full OR gene repertoire in human and mouse with respect to the 17 OR gene families (Figure 1). Moreover, the sample estimates of the fractions of pseudogenes were accurate (see Materials and Methods; Figure 2). This pilot study demonstrates that the degenerate primers yield an unbiased representation of the OR gene repertoire, as measured by the family composition and pseudogene content of the human and mouse samples. Since the primers performed well both in human and a distantly related species, the mouse, there was no reason to assume that they would not do so in nonhuman primate species.

We therefore proceeded to sequence 100 genes from 18 nonhuman primates using these primer pairs. Since the genome sequence is not available for these species, we were not able to compare the familial composition of our samples of OR genes to that of the full OR repertoires. However, with the exception of OR families 3, 11, 12, and 55 (whose absence in a sample of 100 genes is not unlikely, as they represent less than 1.8% of human OR genes), we identified OR genes from all families in all species (Table 1). Moreover, the representation of the three largest OR gene families in the sample varied across species, again suggesting that there is no strong bias towards the amplification of specific families.

**Table 1:**
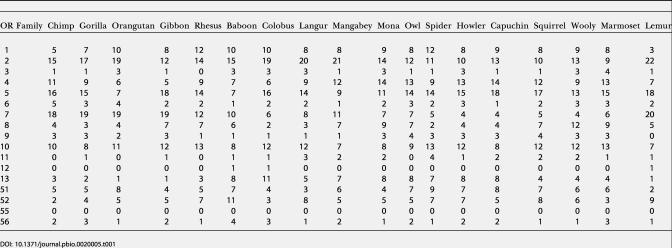
Distribution of OR Genes in Families across Species

We then tabulated the proportion of OR pseudogenes in each species (Figure 2). Consistent with previous results based on direct sequencing of full-length OR orthologs (Gilad et al. 2003), we found that the proportion of OR pseudogene in the great apes and rhesus macaque is approximately 30% (Figure 2). Together, these findings confirm the validity of this degenerate primer approach.

We further found that the proportion of OR pseudogenes in OWMs (29.3% ± 2.4%) is very similar to that of nonhuman apes (33.0% ± 0.8%), but notably higher than that of NWMs (18.4% ± 5.6%). One NWM species, the howler monkey, was a conspicuous exception, with an elevated proportion of OR pseudogenes, similar to that of OWMs and apes (31.0%) (Figure 2) and significantly higher than any other NWM (one-tailed *p* < 0.02 for the difference between the howler monkey and the NWM with the second highest proportion of pseudogenes, the Wooly monkey, as assessed by a Fisher's exact test [FET]). Thus, it appears that a deterioration of the olfactory repertoire occurred in all apes and OWMs as well as, independently, in the howler monkey lineage.

**Figure 3:**
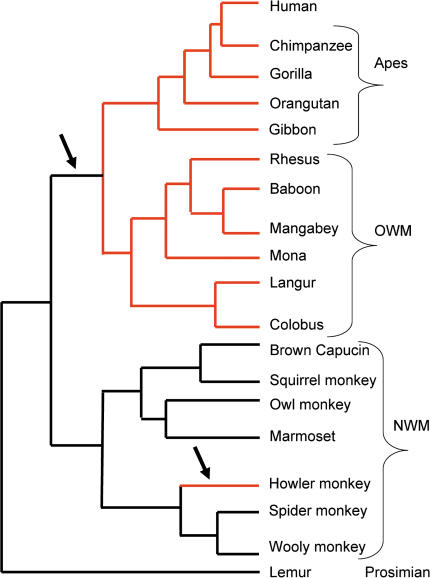
Phylogenetic Tree of Primates Schematic phylogenetic tree of the primate species used in the current study. Phylogenetic relationships between species are based on Harada et al. (1995), Page et al. (1999), and Surridge et al. (2003). Arrows indicate on which lineages the acquisition of full trichromatic color vision occurred (Goodman et al. 1998; Jacobs and Deegan 2001). The red color highlights lineages with a high proportion of OR pseudogenes.

Strikingly, a second phenotype is shared only by the howler monkey, OWMs, and apes: full (or “routine”) trichromatic color vision. In primates, trichromatic color vision is accomplished by three opsin genes whose products are pigments sensitive to short, medium, or long wavelength ranges of visible light (Nathans et al. 1986). In OWMs and apes, the short-wavelength opsin gene is found on an autosome, while two distinct X-linked loci for medium and long wavelengths underlie full trichromatic color vision (and so are present in both males and females). In contrast, most NWM species carry an autosomal gene and only one X-linked gene, where different alleles encode for photopigment opsins that respond to medium or long wavelengths. Heterozygous females can therefore possess trichromatic vision, but males are dichromatic (Jacobs 1996; Boissinot et al. 1998; Hunt et al. 1998). The sole exception among NWMs is the howler monkey (Jacobs et al. 1996; Jacobs and Deegan 2001; Surridge et al. 2003), which has a duplication of the opsin genes on the X chromosome (Goodman et al. 1998; Jacobs and Deegan 2001) (Figure 3). Thus, full trichromatic vision arose twice in primates, once in the common ancestor of OWMs and apes and once in the howler monkey lineage.

While OWMs, apes, and the howler monkey carry 32.5% ± 6.3% OR pseudogenes in their OR gene repertoire, species without full trichromatic vision have 16.7% ± 1.0%, significantly fewer (*p* < 10^−4^, or, excluding humans from the full trichromatic group, *p* < 10^−3^, as assessed by a Mann–Whitney *U* test). This *p* value is only indicative since the species lineages are not all independent. However, if significance is instead assessed by a FET for all pairwise comparisons of species with full trichromatic color vision and without, the difference is again striking: 94 out of 96 comparisons are significant at the 5% level. Thus, the evolution of full trichromatic vision coincided with an increase in the fraction of OR pseudogenes, indicative of a deterioration of the sense of smell.

Apes and OWMs acquired trichromatic color vision approximately 23 million years ago (Yokoyama and Yokoyama 1989), while the duplication of the opsin genes in the howler monkey occurred approximately 7–16 million years ago (Jacobs 1996; Cortes-Ortiz et al. 2003). In spite of this difference in timing, the proportion of OR pseudogenes in species from both lineages is very similar. We estimated the rate of fixation of neutral gene disruptions for OR genes to be approximately 0.12 per gene per million years (Y. Gilad, S. Pääbo, and G. Glusman, unpublished data). This estimate implies that both apes, OWMs and the howler monkey could have a much higher proportion of OR pseudogenes than observed (data not shown), indicating that the process of functional OR gene loss has decreased or stopped in these species. A plausible explanation for the similar proportion of OR pseudogenes in the different lineages is that while full trichromatic vision relaxed the need for a sensitive sense of smell, it did not render olfaction unnecessary. Accordingly, while some OR genes can accumulate coding region disruptions, others are still evolving under evolutionary constraint. This model predicts that the possession of full trichromatic color vision alone allows for the loss of some but not all OR genes. A natural next step would then be to identify which OR genes or families were lost after the acquisition of full trichromatic vision. The answer to this question awaits sequence from a large number of orthologous OR genes.

In this respect, it is interesting to note that the *TRP2* gene, a major component of the vomeronasal pheromone transduction pathway, was found to be intact in several NWM species, but is a pseudogene in OWMs and apes (Liman and Innan 2003; Zhang and Webb 2003). The authors raised the possibility of a connection between the acquisition of full trichromatic color vision and decreased pheromone perception, based on the difference between OWMs and apes on the one hand and NWMs on the other (Liman and Innan 2003; Zhang and Webb 2003). However, since many traits can potentially be mapped to the lineage that leads to OWMs and apes, the connection between full trichromatic vision and pheromone perception was tenuous. Furthermore, Liman and Innan (2003) did not find a coding region disruption in four exons of *TRP2* in the howler monkey. An intact *TRP2* gene in the howler monkey would be inconsistent with the hypothesis that the enhancement of color vision replaced pheromone signaling in primates.

In contrast, in the present study, we find that the deterioration of the olfactory repertoire occurred concomitant with the evolution of full trichromatic vision in two separate primate lineages. Thus, although at this point we are unable to demonstrate that the decline in the sense of smell is a direct result of the evolution of color vision, our results strongly suggest an exchange in the importance of these two senses in primate evolution. Future studies of the sensory cues involved in detection and selection of food (e.g., Smith et al. 2003), or the choice of a mate, may test this association directly.

## Materials and Methods

### 

#### Design and test of degenerate primers. 

OR genes have a coding region that is approximately 1 kb long and contains no introns. In order to test the performance of degenerate primers, we sequenced 30 genes amplified with each primer pair in human and mouse and compared the composition of the different OR families in the sample to that of the full OR gene repertoire of these two species (Glusman et al. 2001; Zhang and Firestein 2002). We also compared the sample estimates of the proportion of pseudogenes to the proportion in the entire OR repertoire of human and mouse. Since the degenerate primers amplify only 670 bp of the approximately 1 kb coding region of the OR gene, a subset of the coding region disruptions will fall in segments of OR genes not amplified by our primers. As a result, the true fraction of OR genes carrying coding region disruptions will be underestimated by our approach. We therefore determined the proportion of OR genes with at least one disruption within the corresponding 670 bp in the entire human and mouse OR gene repertoires (47.7% and 16.3% in humans and mouse, respectively).

We first tested an existing set of primers, used by Rouquier et al. (2000), but found significant deviations from the family composition of the full OR repertoire in both species. As an illustration, among the 60 OR genes obtained in humans, 36.6% were of the subfamily 7E (all pseudogenes), significantly more than expected given the true proportion of the 7E subfamily in the full human OR gene repertoire (12.4%, *p* = 2 × 10^−6^, assessed by FET). As a consequence of these biases, estimates of the proportion of pseudogenes in human and mouse obtained with these primers (Rouquier et al. 2000) differ significantly from the true value (*p* < 0.01, assessed by FET).

We proceeded by designing new pairs of degenerate primers for the OR gene family by using the program HYDEN (Fuchs et al. 2002; Linhart and Shamir 2002). The first primer pair, PC1 (PC1–5′: CTSCAYSARCCCATGTWYHWYTTBCT, PC1–3′: GTYYTSAYDCHRTARAYRAYRGGGTT), was designed based on class 1 human OR sequences only. The second primer pair, PC2 (PC2–5′: YTNCAYWCHCCHATGTAYTTYTTBCT, PC2–3′: TTYCTNARGSTRTAGATNANDGGRTT), was designed based on solely class 2 human OR sequences, excluding all genes that belong to subfamily 7E. Both primer pairs were designed to amplify a 670-bp product that approximately covers the region from transmembrane domains 2–7 of the OR protein. As a first step, we used each primer pair to amplify and sequence (see below) 30 genes from human genomic DNA. We found that PC1 primer pairs amplify OR class 1 and OR class 2 genes in roughly equal proportions. PC2 primer pairs amplified only OR class 2 genes, including members of the 7E OR subfamily. Based on the OR family composition that we observed for the 60 genes, we estimated that if we constructed a sample containing 25% of genes amplified with PC1 and 75% of genes amplified with PC2, we would obtain an unbiased representation of the familial composition of the human OR gene repertoire. This approach was validated by amplifying and examining 100 genes collected in the same way from human as well as from mouse.

#### PCR and DNA sequencing

Each primer pair was used to amplify a set of eight reactions in each species using a temperature-gradient PCR. The use of several annealing temperatures for each species yielded a greater diversity of amplified OR genes. PCR was performed in a total volume of 25 μl, containing 0.2 μM of each deoxynucleotide (Promega, Madison, Wisconsin, United States), 50 pmol of each primer, 1.5 mM MgCl_2_, 50 mM KCl, 10 mM Tris (pH 8.3), 2 U of Taq DNA polymerase, and 50 ng of genomic DNA. Conditions for the PCR amplification from all species were as follows: 35 cycles of denaturation at 94°C, annealing at a gradient temperature of 48°C to 60°C, and extension at 72°C, each step for 1 min. The first step of denaturation and the last step of extension were 3 min each. The PCR products were separated and visualized in a 1% agarose gel. From each amplification set (a given primer pair in a given species), all successful products were mixed and subjected to cloning using a TA cloning kit (Boehringer, Mannheim, Germany). Cloning was followed by a touchdown PCR using the vector primers for amplifications from isolated bacterial colonies. Products were purified using the High Pure PCR Product Purification Kit (Boehringer). Sequencing reactions were performed in both directions on PCR products, using the vector primers and the dye-terminator cycle sequencing kit (Perkin Elmer, Wellesley, Massachusetts, United States) on an ABI 3700 automated sequencer (Perkin Elmer).

#### Sequence analysis

After base calling with the ABI Analysis Software (version 3.0), the data were edited and assembled using the Sequencher program, version 4.0 (GeneCodes Corporation, Ann Arbor, Michigan, United States). Assembly of the clones was done using a similarity cutoff of 98%. This cutoff ensures that Taq-generated mutations that may have been sequenced in individual clones are not counted as independent genes. Clones that were collapsed to the same contig by the assembly process were counted as one gene. Once 25 and 75 genes (independent contigs) were identified from PC1 and PC2 primer pairs, respectively, a majority consensus was generated for each gene. In order to confirm that only OR genes were amplified from all the species, we used the consensus sequences of all genes from all species as queries in a BLAT search against the human genome sequence (http://genome.ucsc.edu/). In every case, the best hit was a human OR gene. This analysis was also used to insure that none of the genes were an artifact of (“jumping”) PCR fusion. Finally, each consensus sequence was searched for an uninterrupted open reading frame (ORF) in all six possible frames. If an uninterrupted ORF was found, the gene was annotated as intact. If no ORF was identified, the gene was annotated as a pseudogene. This approach probably results in an underestimate of the proportion of pseudogenes, as not all OR genes with an intact coding region are functional. Mutations in promoter or control regions of OR genes may lead to reduced or no expression. Similarly, radical missense mutations in highly conserved positions of the OR protein may result in dysfunction (Menashe et al. 2003). Although it is known that there are several highly conserved positions among OR genes, it is not always straightforward to ascertain which, if any, of these positions is necessary to retain function. We therefore chose the most straightforward definition of a pseudogene: a gene without a full ORF.

## Supporting Information

### Accession Numbers

Sequences for all OR genes from all primate species were deposited to GenBank (http://www.ncbi.nlm.nih.gov/Genbank/) as accession numbers AY448037–AY449380 and AY454789–AY455274.

## Abbreviations

FET: Fisher's exact test
OR: olfactory receptor
ORF: open reading frame
OWM: Old World monkey
NWM: New World monkey
