# Correction: Loss of Olfactory Receptor Genes Coincides with the Acquisition of Full Trichromatic Vision in Primates

**DOI:** 10.1371/journal.pbio.0050148

**Published:** 2007-06-12

**Authors:** Yoav Gilad, Victor Wiebe, Molly Przeworski, Doron Lancet, Svante Pääbo

In *PLoS Biology*, volume 2, issue 1: doi: 10.1371/journal.pbio.0020005

The Max Planck Society has requested that the following two statements be published:

**Statement 1: Erratum**

Y. Gilad, M. Przeworski, and D. Lancet

Upon reanalysis of these data, we found a number of mistakes and realized that salient details were missing from the Materials and Methods section. In particular, once we corrected mistakes in the annotation, we found that the total number of distinct genes that were sequenced from each species actually ranged from 89 to 100. In the analysis, we excluded 7E sequences in apes that differed by more than 25 base pairs from the expected size. Moreover, while the vast majority (>97%) of sequences with more than 98% identity were collapsed, multiple sequences were treated as distinct genes when they clearly did not represent random taq errors. The exact sequences used in the analysis are available from http://rd.plos.org/pbio.0050148. These mistakes do not change the qualitative conclusions, but some of the numbers have changed: The corrected proportion of OR pseudogenes in non-human apes is 36 ± 2.9%, in OWM it is 32 ± 4.3%, and in NWM it is 18.0 ± 1.4%. The proportion of pseudogenes in the howler monkey remains 31%.

**Statement 2**

V. Wiebe and S. Pääbo retract their names from the publication since they are of the opinion that the primary data do not support the conclusions presented.

